# S100A9 blockade prevents lipopolysaccharide-induced lung injury via suppressing the NLRP3 pathway

**DOI:** 10.1186/s12931-021-01641-y

**Published:** 2021-02-06

**Authors:** Boying Zhao, Renfu Lu, Jianjun Chen, Ming Xie, Xingji Zhao, Lingwen Kong

**Affiliations:** 1 Department of Cardiothoracic Surgery, Chongqing Emergency Medical Center, Chongqing University Central Hospital, Chongqing University, No. 1 Jiangkang Road, Yuzhong, Chongqing, 400010 China; 2 Vascular Surgery Department, The First Affiliated Hospital of Chongqing Medical University, Chongqing, China

**Keywords:** S100A9, Inflammatory alarmin, Acute lung injury, NLRP3

## Abstract

**Background:**

S100 calcium binding protein A9 (S100A9) is a pro-inflammatory alarmin associated with several inflammation-related diseases. However, the role of S100A9 in lung injury in sepsis has not been fully investigated. Therefore, the present study aimed to determine the role of S100A9 in a lipopolysaccharide (LPS)-induced lung injury murine model and its underlying molecular mechanisms.

**Methods:**

LPS was utilized to induce sepsis and lung injury in C57BL/6 or NOD-like receptor family pyrin domain containing 3 (NLRP3)^−/−^ mice. To investigate the effects of S100A9 blockade, mice were treated with a specific inhibitor of S100A9. Subsequently, lung injury and inflammation were evaluated by histology and enzyme‑linked immunosorbent assay (ELISA), respectively. Furthermore, western blot analysis and RT-qPCR were carried out to investigate the molecular mechanisms underlying the effects of S100A9.

**Results:**

S100A9 was upregulated in the lung tissues of LPS-treated mice. However, inhibition of S100A9 alleviated LPS-induced lung injury. Additionally, S100A9 blockade also attenuated the inflammatory responses and apoptosis in the lungs of LPS-challenged mice. Furthermore, the increased expression of NLRP3 was also suppressed by S100A9 blockade, while S100A9 blockade had no effect on NLRP3^−/−^ mice. In vitro, S100A9 downregulation mitigated LPS-induced inflammation. Interestingly, these effects were blunted by NLRP3 overexpression.

**Conclusion:**

The results of the current study suggested that inhibition of S100A9 could protect against LPS-induced lung injury via inhibiting the NLRP3 pathway. Therefore, S100A9 blockade could be considered as a novel therapeutic strategy for lung injury in sepsis.

## Introduction

Sepsis is a fatal disease, characterized by multiple organ failure. The lung is the most vulnerable organ during sepsis [1]. Serious lung injury may lead to acute respiratory distress syndrome (ARDS) and respiratory failure. The mortality rate of these diseases is estimated to be 35–45% in intensive care units [2]. Currently, there is no specific intervention for the treatment of sepsis-mediated lung injury and ARDS. Therefore, novel therapeutic targets are urgently needed to improve the prognosis of patients with sepsis-mediated lung injury.

Trauma and infection are the main causes of sepsis and related lung injury. Both conditions can trigger excessive inflammatory responses in the lung [3]. A growing body of studies have suggested that S100 calcium binding protein A9 (S100A9) and its dimerization partner S100A8, also known as myeloid-related proteins 8 and 14, play critical role in inflammation and inflammation-related tissue damage [4, 5]. Approximately 40% of the cytosolic proteins in neutrophils are constituted by S100A9. Therefore, S100A9 is mainly stored in neutrophils and rapidly released upon activation [6]. Other inflammatory cells, including monocytes and macrophages, also produce S100A9 in small amounts [7]. It has been reported that S100A9 can induce immune cell activation via the Toll-like receptor 4 (TLR4) or advanced glycation end products (RAGE) inflammatory signaling pathways [8, 9]. Excessive expression of S100A9 has been observed in several infection- and non-infection-induced inflammatory diseases, such as sepsis, myocardial infraction and chronic obstructive pulmonary disease [10–12]. It has been also reported that S100A8/A9 promote lung injury via the TLR4-dependent activation of alveolar epithelial cells [13]. Furthermore, recent studies have also shown that S100A9 is associated with poor outcomes in coronavirus disease 2019 (COVID-19)-induced pneumonia [14]. In addition, several studies have suggested that S100A9 downregulation exerts beneficial effects on inflammatory diseases [15]. However, the effects of S100A9 blockade in lipopolysaccharide (LPS)-induced lung injury have not been previously investigated.

NOD-like receptor family pyrin domain containing 3 (NLRP3) is one of the most important mediators of inflammation [16]. Upon infection, NLRP3 assembles along with caspase-1 and apoptosis-related speck-like protein (ASC) to form inflammasomes, thus inducing inflammatory responses [17]. Inflammatory cells, especially neutrophils and macrophages, infiltrate into the lung tissues [18]. These inflammatory cells induce the secretion of inflammatory cytokines and trigger lung injury [19]. Since S100A9 is mainly stored in neutrophils, blocking its activation may attenuate the NLRP3 pathway-mediated inflammation, and may be, therefore, considered as an efficient therapy for LPS-induced lung injury.

## Methods

### Experimental animal and treatment

All animal experimental procedures were approved by the Animal Care and Use Committee of Chongqing University, and were in accordance with the National and Institutional Guidelines for Animal Care. Ten-week-old male C57BL/6 mice were provided by the Experimental animal Center of Chongqing Medical University. NLRP3^−/−^ mice with C57BL/6 background were obtained from the Key Laboratory of Child Development and Diseases of Children’s Hospital of the Chongqing Medical University. Mice were housed at a temperature of 21–22 °C and relative humidity of 50% with a 12-h light/dark cycle. Animals were treated with 5 mg/kg LPS in sterile saline to induce lung injury, as described previously [20]. Additionally, mice in the control group received sterile saline alone. To investigate the effects of S100A9 blockade, mice were intraperitoneally injected with ABR-238901 (MedchemExpress), a quinoline-3-carboxamide analog and specific inhibitor of S100A9, at doses of 20 or 30 mg/kg in dimethyl sulfoxide (DMSO) simultaneously with LPS administration. Mice in the control and LPS groups were treated with equal amount of DMSO [21]. Depending on the experiment, all mice were anesthetized with 2 or 3% isoflurane, for induction and maintenance, respectively. When needed, mice were sacrificed with inhalation of 5% isoflurane followed by cervical dislocation at the indicated time points to collect the lung tissues.

### Lung wet-to-dry ratio

Pulmonary edema was evaluated by lung wet to dry wet ratio as described previously [22]. Briefly, lungs were harvested and weighted for wet weight. Then the lung was dehydrated in incubator at 80 ℃ for 24 h to get dry weight for calculating Wet-to-dry ratio.

### Bronchoalveolar lavage fluid acquisition (BALF) and analysis

Mice were treated with heparin and anesthetizing. A small incision in the trachea was made with the tips of forceps. Then 3 mL cold PBS was injected into lungs through the incision, the fluid was sucked out after massaging the chest. Repeating this perfusion procedure for three times and collected about 1.5 mL fluid. The collected fluid was subsequently centrifuged at 1000 rpm for 10 min at 4℃. The protein content in BALF was evaluated with a BCA assay kit.

### Inflammatory cytokines and S100A9 concentration measurements

The levels of TNF-α and IL-1β in BALF as well as medium were detected with TNF-α and IL-1β Elisa kit (Beijing 4A Biotech Co., Ltd) according to the manufacturer’s instructions. The concentration of S100A9 in BALF and lung homogenates was measured with a mouse S100A9 ELISA kit (Yuanmu Biotech Co., Ltd) according to the manufacturer's instruction.

### Measurement of myeloperoxidase (MPO) and lactate dehydrogenase (LDH) activity

Lung homogenates was used for evaluating the MPO activity and LDH content with corresponding assay kit (Jiangcheng Biotechnology, China) according to the manufacturer's instruction.

### Measurement of caspase-3 activity

The caspase-3 activity was measured with Caspase 3 Activity Assay Kit (Beyotime, China) according to the manufacturer's instruction. The lung tissues were homogenized in lysis buffer. After centrifugation at 16000*g* for 15 min at 4 ℃, the supernatant was collected. Then the lysates were incubated with Ac-DEVD-pNA at 37℃ for 100 min before detected.

### Histological analysis

Mice were treated with heparin and anesthetized with 2% isoflurane. Subsequently, the left lung was harvested and fixed in 4% paraformaldehyde. Tissues were embedded in paraffin and cut into 7-μm-thick sections for hematoxylin and eosin (H&E) staining. The tissue pathology was assessed based on the inflammatory cell infiltration, edema degree and the thickness of alveolar septum in at least five random fields under a light microscope (magnification, × 200) [20]. Briefly, based on the proportion of the damaged area sections were rated on a 0–5 scale. The scoring system was as follows: 0 points, no damage; 1 point, injury covering 20% of the field; 2.5 points, injury covering 50% of the field; and 5 points, injury covering 100% of the field.

### RT-PCR

Total RNA form lung tissue was extracted using Trizol regent (TaKara, Japan), 1000 ng RNA was reverse transcribed with Superscript. Subsequently, qRT-PCR was performed with SYBR green reagent on Bio-rad real time PCR system. Primers used for measuring gene expression were shown below: IL-1β(forward, TGACAGTGAGAATGACCTGTTC; reverse, TTGGATGACCCTCTTAHTHTTC), TNF-α (forward, ACAGCAAGGGACTAGCCAGGAG; reverse, GGAGTGCCTCTTCTGCCAGT) and β-actin ( forward CCCTAAGGCCAACCGTGAAA; reverse ACGACCAAGGCATACAGGGA).

### Cell culture and transfection

The human lung epithelia cells line, BEAS-2B, was purchased from China Cell Line Bank. The cells were maintained in DMEM with 10% fetal bovine serum (Every Green, China), 0.022% sodium pyruvate and 0.26% sodium bicarbonate. To overexpress NLRP3, cells were transfected with plasmids encoding NLRP3 (Addgene, USA) by Lipofectamine 3000 (Thermo Fisher, USA) for 24 h. Then the cells were treated with 100 ng/ml LPS for 8 h followed by 5 mM ATP for 1 h, with or without 10 μM ARB starting at 24 h after transfection.

### Western blot analysis

Heart tissues or cells were lysed with RIPA lysis buffer (Beyotime Institute of Biotechnology), and the protein extracts were then centrifugated at 12,100 rpm for 15 min. Subsequently, the protein concentration was measured with a BCA Protein Assay. A total of 30 μg protein extracts were separated by SDS-PAGE and were then electrotransferred onto polyvinylidene fluoride membranes. Following blocking with 5% non-fat milk for 1 h at room temperature, the membranes were first probed with primary antibodies overnight at 4 °C, and then with the corresponding secondary antibodies. The protein blots were visualized using an ECL imaging system (Bio-Rad Laboratories, Inc.).

### Co-immunoprecipitation

Lung tissues were lysed with NP-40 (Beyotime, China) followed by centrifugation at 12,000 RPM for 15 min at 4 ℃ and the supernatant was collected. protein concentration were measured by BCA protein assay kit (Beyotime, China). Then 200 μg lysates were incubated with Protein A magnetic Beads (Cell signaling technology, USA) with a tube rotator at 100 revolutions per minute for 2 h at 4 °C. After the beads were discarded by magnetic force, the antibody of S100A9 (Abcam, ab105472, 1:50) was added to the precleared lysate at 4 °C for overnight. After aspirating the supernatant, the complex were washed with NP-40 buffer with 500 nM NaCl for 5 times and then resuspended in sample buffer, followed by incubation at 98 °C for 10 min to release the proteins form beads. Then the protein was used for western blot to detect the interaction of S100A9 and TLR4.

### Statistical analysis

All values are expressed as the mean ± standard error of the mean (SEM). Data were analyzed with one-way or two-way analysis of variance (ANOVA) followed by Tukey’s multiple comparisons test. Survival curves were constructed using the Kaplan–Meier survival curve and analyzed using a log-rank test. P < 0.05 was considered to indicate a statistically significant difference.

## Results

### S100A9 is upregulated in the lungs of LPS-treated mice

**Fig. 1:**
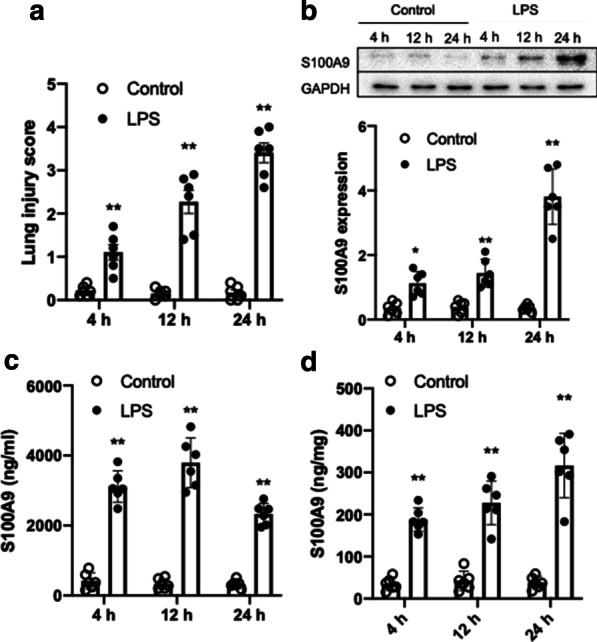
Effects of LPS on lung injury and S100A9 expression in mice. Mice were treated with LPS (5 mg/kg) and the lung tissues and BALF were collected at 4, 12 and 24 h following LPS treatment. **a** LPS induced lung injury starting at 4 h. **b** The protein expression levels of S100A9 in the lung tissues of mice are shown. The semi-quantified results of S100A9 expression are also shown. **c** The concentration of S100A9 in BALF is presented. **d** The concentration of S100A9 in lung homogenates is presented. Data are expressed as the mean ± SEM (n = 6 for each group). Statistical analysis was performed using two-way ANOVA followed by Tukey's multiple comparison test. ^**^P < 0.01 vs. the corresponding control. *LPS* lipopolysaccharide, *S100A9* S100 calcium binding protein A9, *BALF* bronchoalveolar lavage fluids

Firstly, the degree of lung injury and the expression of S100A9 in the lung tissues of LPS-treated mice was measured at 4, 12 and 24 h following treatment with LPS. The results showed that LPS significantly induced lung injury starting at 4 h (Fig. 1a). Consistent with lung injury, the expression of S100A9 was upregulated in a time-dependent manner and reached to approximately eightfold increase at 24 h after stimulation with LPS (Fig. 1b). Subsequently, the concentration and expression of S100A9 were determined in bronchoalveolar lavage fluids (BALF) and lung tissues from LPS-treated mice, respectively, at the indicted time points. Compared with the control group, the levels of S100A9 were significantly increased in BALF (Fig. 1c) and lung homogenates (Fig. 1d) at 4 h following LPS treatment. These findings suggested that the expression of S100A9 was acutely elevated at the early stage of LPS-induced lung injury.

### S100A9 blockade ameliorates LPS-induced lung injury in mice

**Fig. 2:**
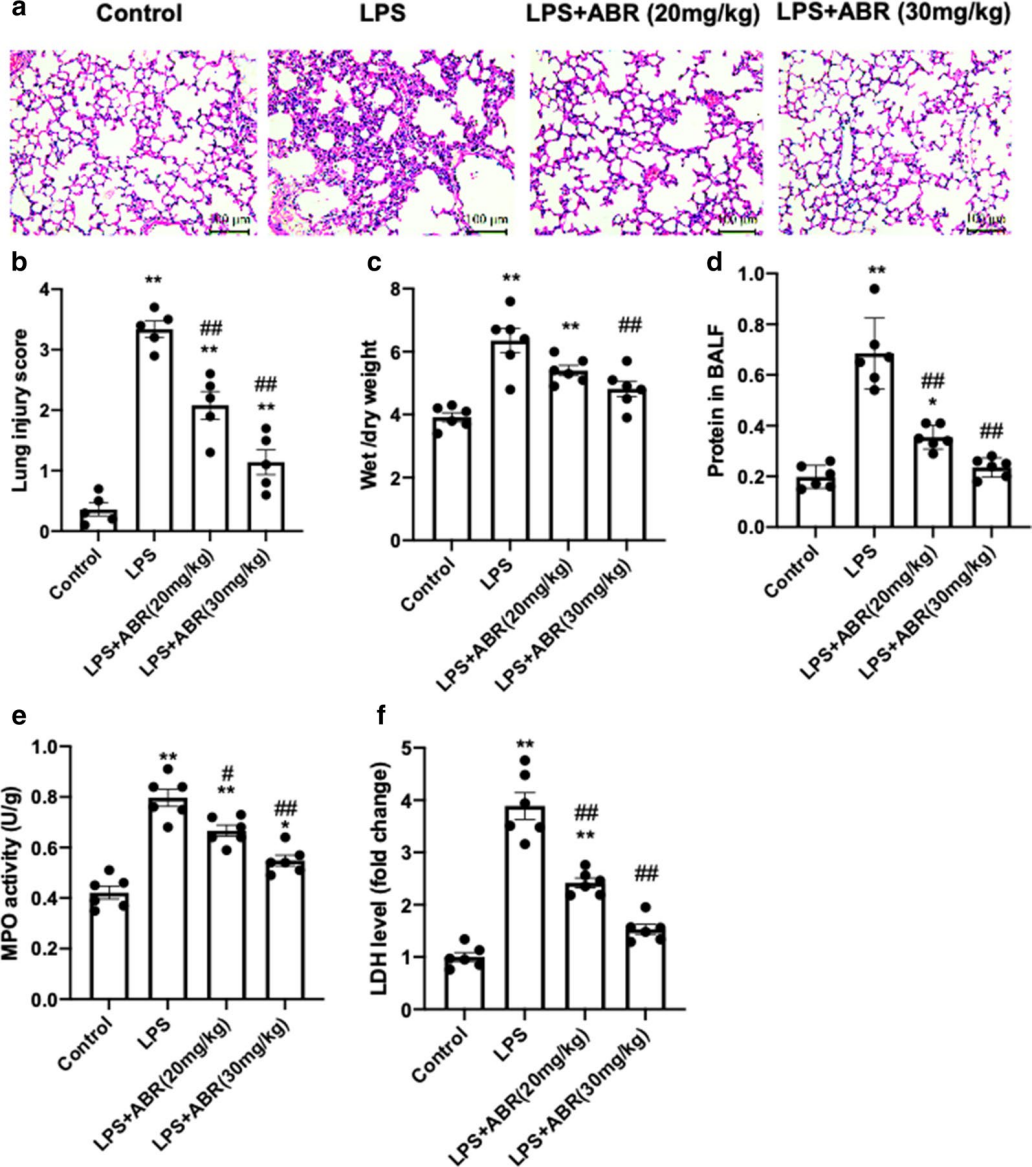
S100A9 blockade ameliorates lung injury in LPS-treated mice. **a** Representative images of hematoxylin and eosin staining of the left lung (scale bar, 100 μm). **b** The lung injury score was determined based on histological analysis. **c** The lung wet-to-dry weight ratio is presented. **d** The total protein levels in BALF were measured using a BCA assay. **e**–**f** The MPO activity and LDH content in lungs were measured to evaluate oxidative injury. Data are expressed as the mean ± SEM (n = 6 for each group). Statistical analysis was performed using one-way ANOVA followed by Tukey's multiple comparison test. ^*^P < 0.05, and ^**^P < 0.01 vs. the control group; ^#^P < 0.05, ^##^P < 0.01 vs. the LPS group. *S100A9* S100 calcium binding protein A9, *LPS* lipopolysaccharide, *BALF* bronchoalveolar lavage fluids, *MPO* myeloperoxidase, *LDH* lactate dehydrogenase

To evaluate whether S100A9 serves a pathogenic role in LPS-induced lung injury, the effects of S100A9 blockade on lung injury were determined. Therefore, mice were co-treated with 20 or 30 mg/kg of the specific S100A9 blocker, ABR, and LPS. Following treatment for 24 h, the lung tissues and BALF were collected. Compared with the control group, treatment with LPS resulted in significant lung injury, evidenced by increased inflammatory cell infiltration (Fig. 2a), lung injury score (Fig. 2b), wet/dry weight ratio (Fig. 2c) and BALF protein content (Fig. 2d). However, inhibition of S100A9 with ABR notably mitigated the LPS-induced lung injury (Fig. 2a–d). Therefore, the inflammatory cell infiltration, lung injury score, wet/dry weight ratio and BALF protein content were restored. In addition, the enhanced myeloperoxidase (MPO) activity and increased lactate dehydrogenase (LDH) levels in lung tissues of LPS-treated mice were also remarkably attenuated by S100A9 blockade (Fig. 2e, f). The aforementioned results indicated that S100A9 blockade alleviated LPS-induced lung injury in mice.

### S100A9 blockade attenuates inflammatory responses and apoptosis in the lung tissues of LPS-treated mice

**Fig. 3:**
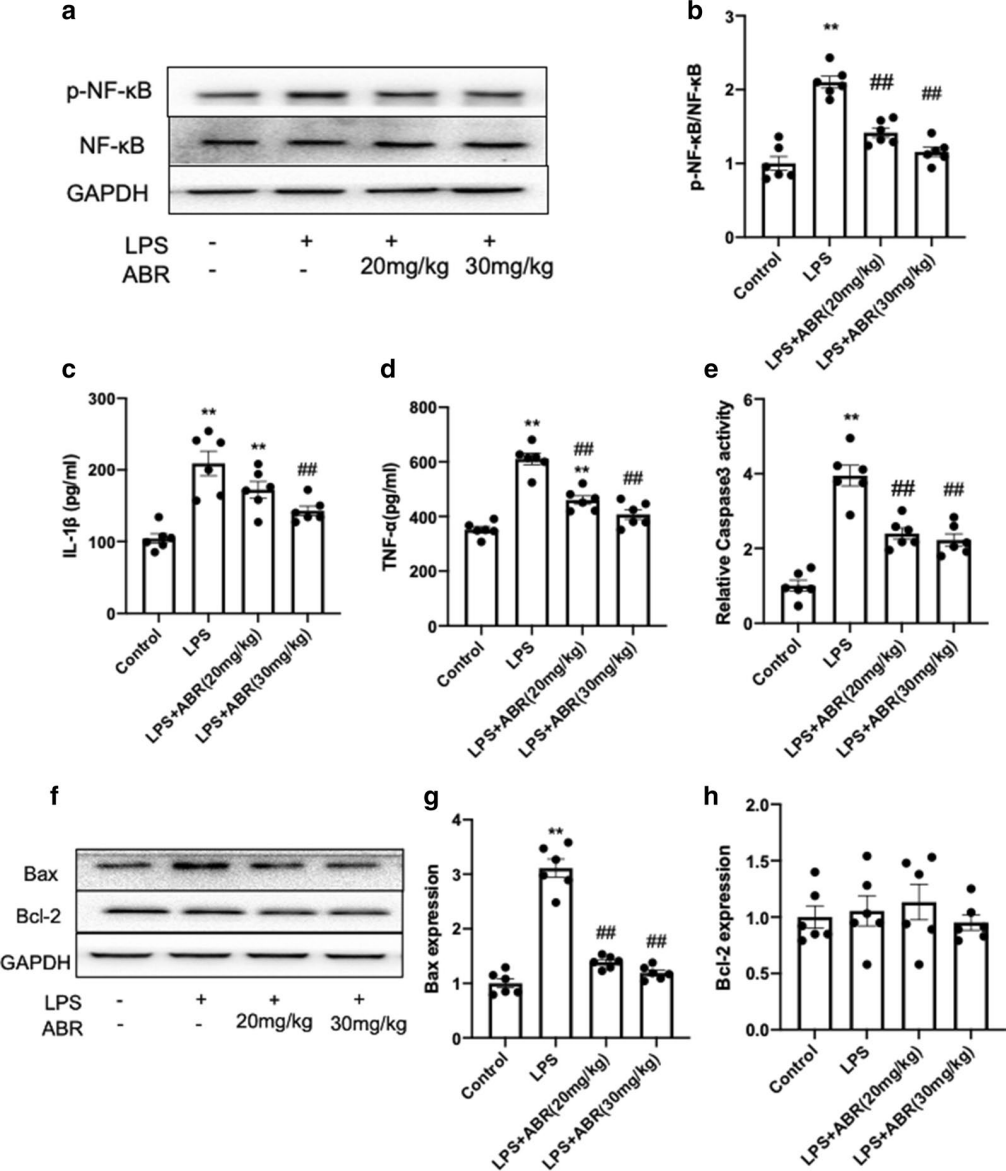
S100A9 blockade attenuates inflammatory response and apoptosis in lungs. **a**, **b** S100A9 blockade attenuated the activation of NF-κB in the lungs of LPS-treated mice. **c**, **d** The secretion levels of TNF-α and IL-1β in BALF were measured using an ELISA kit. **e** The caspase-3 activity was determined using an assay kit. **f**–**h** The protein expression levels of Bcl-2 and Bax in the lungs were determined by western blot analysis. The semi-quantified results are shown in g and h. Data are expressed as the mean ± SEM (n = 6 for each group). Statistical analysis was carried out using one-way ANOVA followed by Tukey's multiple comparison test. ^**^P < 0.01 vs. the control group; ^##^P < 0.01 vs. the LPS group. *S100A9* S100 calcium binding protein A9, *NF-κB* nuclear factor κB, *LPS* lipopolysaccharide, *TNF-α* tumor necrosis factor α, *IL-1β* interleukin 1β, *BALF* bronchoalveolar lavage fluids

S100A9 is mainly produced and sorted in neutrophils and macrophages, and S100A9 is considered as an important inflammatory alarmin for triggering immune responses [23]. Subsequently, the present study evaluated the effects of S100A9 blockade on inflammatory responses in the lungs. The results demonstrated that LPS activated the nuclear factor κB (NF-κB) pathway to promote inflammation. However, S100A9 blockade significantly suppressed the activation of NF-κB (Fig. 3a, b). Compared with the control group, the secretion levels of interleukin 1β (IL-1β) and tumor necrosis factor α (TNF-α) were markedly increased in BALF of LPS-treated mice, while inhibition of S100A9 could notably reduce the increased levels of the inflammatory factors (Fig. 3c, d). Furthermore, stimulation with LPS increased cell apoptosis in the lung tissues as evidenced by the increased caspase-3 activity and Bax expression. However, S100A9 blockade significantly blunted LPS-induced apoptosis (Fig. 3e–h). These findings revealed that S100A9 blockade could attenuate the inflammatory responses and apoptosis induced by LPS, thus possibly mitigating lung injury.

### S100A9 blockade suppresses NLRP3 activation in the lungs

**Fig. 4:**
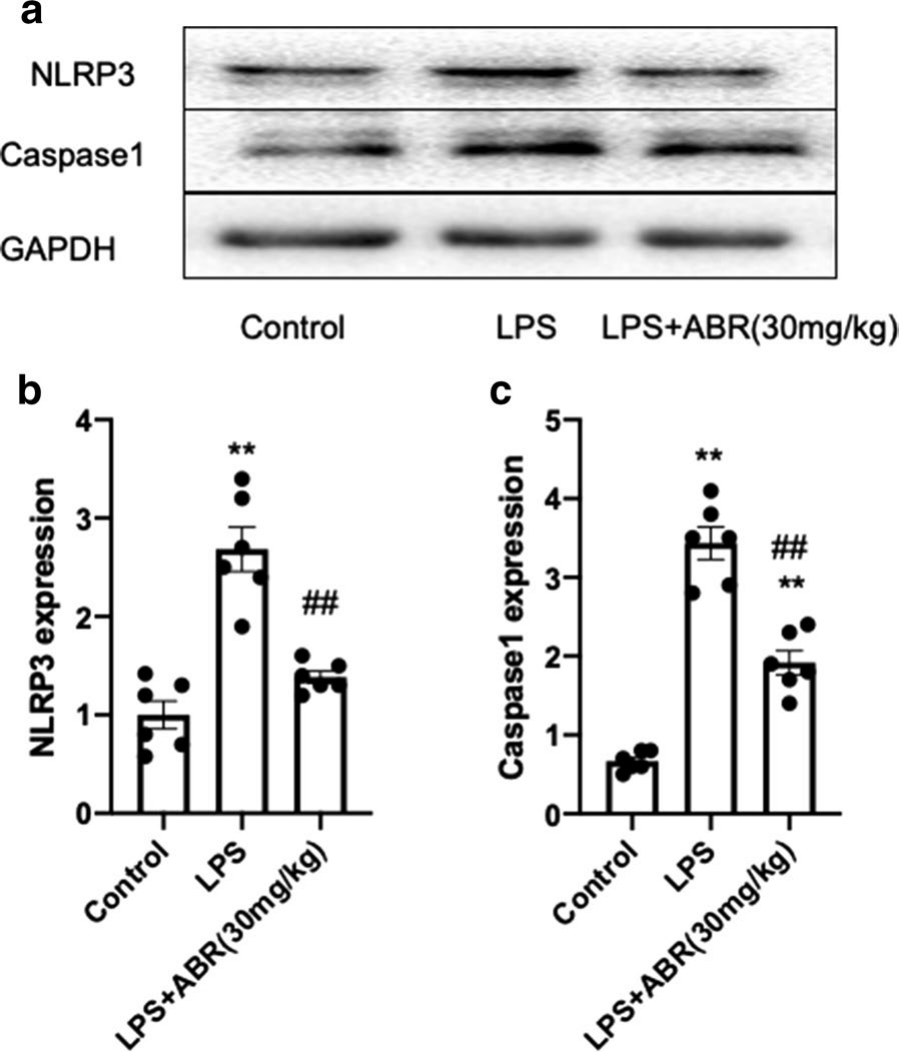
S100A9 blockade suppressed the activation of NLRP3 in the lungs. **a**–**c** Representative images and analysis of the protein expression of NLRP3 and caspase-1 in the lung tissues of LPS-treated mice. Data are expressed as the mean ± SEM (n = 6 for each group). Statistical analysis was carried out using one-way ANOVA followed by Tukey's multiple comparison test. ^**^P < 0.01 vs. the control group; ^##^P < 0.01 vs. the LPS group. *S100A9* S100 calcium binding protein A9, *NLRP3* NOD-like receptor family pyrin domain containing 3, *LPS* lipopolysaccharide

It has been reported that another member of the S100 family, S100A12, promotes sepsis-induced ARDS via activating the NLRP3 pathway [24]. In line with the previous findings, the current study revealed that the increased expression of the NLRP3 inflammasome and caspase-1 were significantly decreased following inhibition of S100A9 in the lung tissues of LPS-treated mice (Fig. 4a–c).This finding further supported the inhibitory effect of S100A9 blockade on inflammatory responses. Overall, these results indicated that inhibition of S100A9 could protect against lung injury via inhibiting the NLRP3-pyroptosis pathway.

### Inhibition of S100A9 mitigates the LPS-induced inflammatory cytokine secretion via the NLRP3 pathway in vitro

**Fig. 5:**
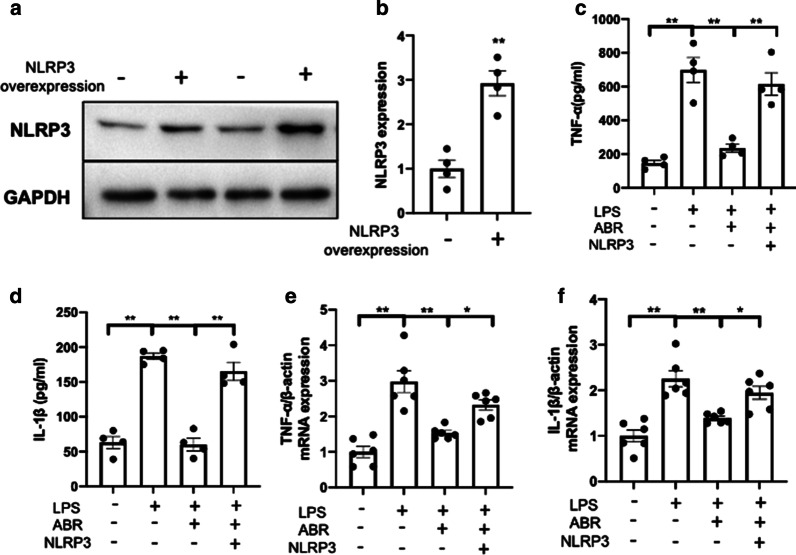
Inhibition of S100A9 mitigates the LPS-induced cytokine secretion via the NLRP3 pathway in vitro. **a**, **b** The NLRP3 overexpression efficiency was evaluated by western blot analysis at 24 h following transfection. **c**-, **d** S100A9 blockade attenuated the secretion of TNF-α and IL-1β via the NLRP3 pathway. **e**, **f** The mRNA expression levels of TNF-α and IL-1β were determined by RT-PCR. Data are expressed as the mean ± SEM (n = 4–6 for each group). Statistical analysis was carried out using one-way ANOVA followed by Tukey's multiple comparison test. *S100A9* S100 calcium binding protein A9, *LPS* lipopolysaccharide, *NLRP3* NOD-like receptor family pyrin domain containing 3, *TNF-α* tumor necrosis factor α, *IL-1β* interleukin 1β

To investigate whether S100A9 blockade could act via suppressing the NLRP3 pathway, the lung epithelial cells, BEAS-2B, were transfected with NLRP3 overexpression plasmids. The transfection efficiency was evaluated by western blot analysis (Fig. 5a, b). Subsequently, BEAS-2B cells were treated with 100 ng/ml LPS for 8 h, followed by co-treatment with 5 mM ATP with or without 10 μM ARB for 1 h. S100A9 inhibition significantly reduced the secretion (Fig. 5c, d) and transcription (Fig. 5e, f) of the inflammatory cytokines, TNF-α and IL-1β, in LPS-stimulated cells. However, NLRP3 overexpression almost completely weakened the effects of S100A9 blockade (Fig. 5a–d). These data suggested that S100A9 inhibition could restore LPS-induced inflammatory responses via the NLRP3 pathway.

### S100A9 blockade has no effect on NLRP3^−/−^ mice

**Fig. 6:**
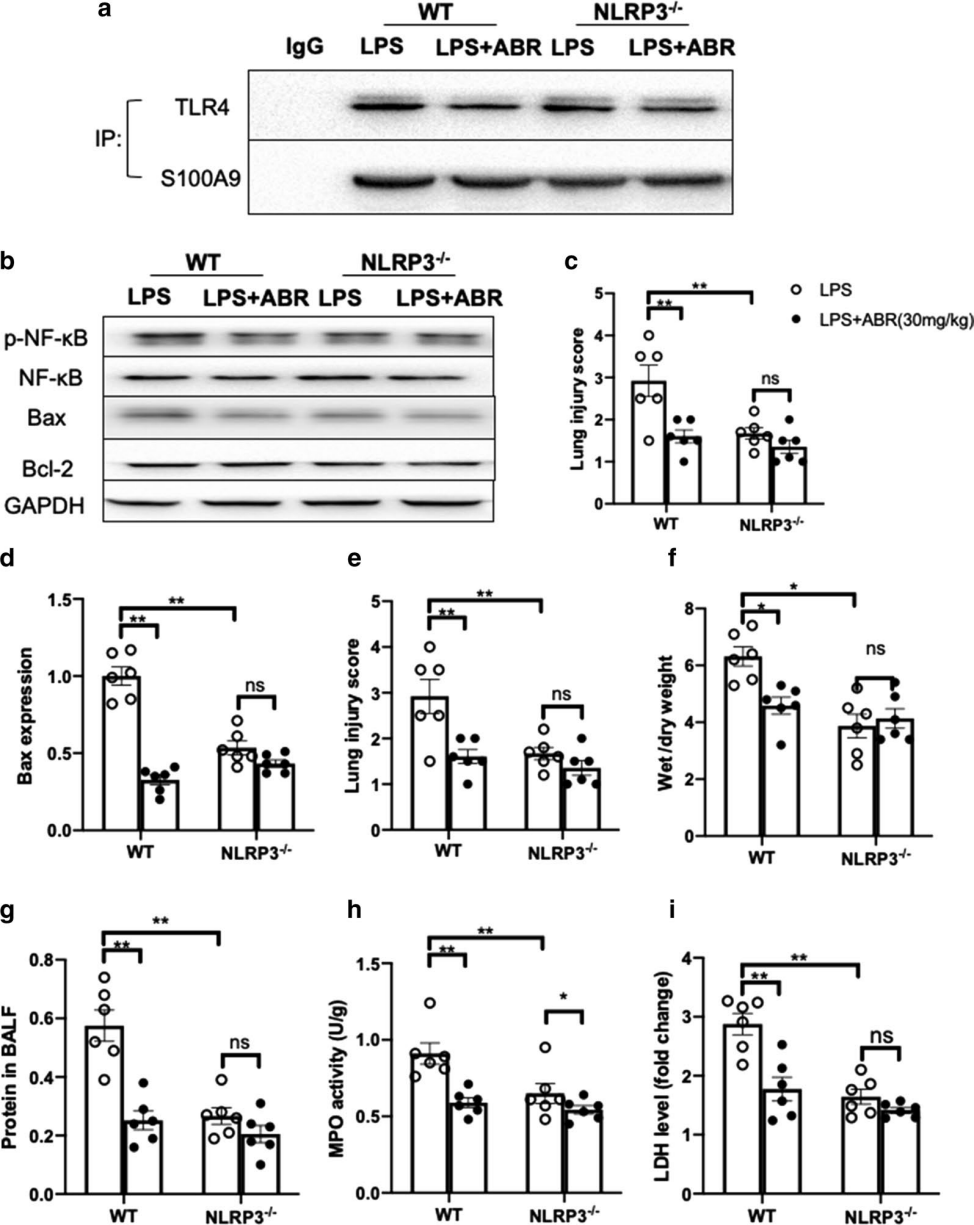
S100A9 blockade exerts its protective effects via inhibiting the NLRP3 pathway. **a** Co-immunoprecipitation was performed to detect the interaction of S100A9 with its receptor (TLR4) in the lung tissues of LPS-treated mice. **b** The protein expression levels of NF-κB and apoptosis-related proteins were measured by western blot analysis. **c**, **d** The quantified results are shown. **e** The lung injury score was determined based on histological analysis. **f** The lung wet-to-dry weight ratio is presented. **g** The total protein levels in BALF are shown. **h**, **i** The MPO activity and LDH levels in the lungs of mice are shown. Data are expressed as the mean ± SEM (n = 6 for each group). Statistical analysis was performed using two-way ANOVA followed by Tukey's multiple comparison test. ^*^P < 0.05, ^**^P < 0.01 vs. the control group. *S100A9* S100 calcium binding protein A9, *NLRP3* NOD-like receptor family pyrin domain containing 3, *TLR4* toll-like receptor 4, *LPS* lipopolysaccharide, *NF-κB* nuclear factor κB, *BALF* bronchoalveolar lavage fluids, *MPO* myeloperoxidase, *LDH* lactate dehydrogenase

To further verify whether S100A9 blockade could act via blocking the NLRP3 pathway in vivo, the effects of S100A9 inhibition were determined in wild-type and NLRP3 null mice. The results showed that genetic silencing of NLRP3 could not affect the interaction of S100A9 with TLR4 in the lung tissues of LPS-treated mice, suggesting that NLRP3 was not the upstream molecule of S100A9 (Fig. 6a). In addition, S100A9 blockade suppressed the activation of NF-κB and downregulated Bax in the lungs of wild-type LPS-treated mice, while these effects were blunted in NLRP3^−/−^ mice (Fig. 6b–d). More importantly, NLRP3 knockout itself could protect against LPS-induced lung injury, while S100A9 blockade could not provide additional protective effects in NLRP3 null mice (Fig. 6e–g). Although the LDH levels were similar between wild-type and NLRP3 null mice, the MPO activity was furtherly decreased by S100A9 blockade in the lungs of LPS-treated NLRP3^−/−^ mice (Fig. 6h, i). This finding indicated that the effect of S100A9 blockade on attenuating oxidative stress was not fully mediated by NLRP3. Collectively, the aforementioned data supported that S100A9 blockade could exert its protective effects via partially inhibiting the NLRP3 pathway.

## Discussion

LPS-induced lung injury is characterized by excessive inflammation and accumulation of inflammatory cells, especially neutrophils and macrophages, in the lungs [25]. Neutrophils are abundant and rapidly responding cells, which are activated and recruited into the injury sites within minutes following exposure of the organism to injury stimuli [26]. It is estimated that 90% of the cell populations in the alveolar spaces consists of macrophages [27]. Activated neutrophils and macrophages produce large amounts of inflammatory factors and mediators, which in turn induce tissue damage and promote inflammation. Therefore, neutrophils and macrophages are considered as the key inflammatory cells in the pathogenesis of LPS-induced lung injury [28, 29]. S100A9 is a Ca^2+^ binding protein, which is constitutively stored in neutrophils and monocytes as a Ca^2+^ sensor [29]. Upon injury or infection, S100A9 is abundantly released and participates in the recruitment of leukocytes and cytokine secretion, which in turn play critical role in modulating inflammation and tissue injury [30]. It has been reported that blockade of S100A9 with small-molecular inhibitors or antibodies alleviates inflammation-related injury, such as septic liver injury and influenza A virus infection-mediated lung injury [16, 31].

Herein, LPS-induced lung injury resulted in increased S100A9 levels in the lung tissues and BALF. To investigate the functional roles of S100A9 in LPS-induced lung injury, mice were treated with a S100A9 inhibitor, at 0 and 24 h following LPS administration. The results showed that the pharmacological inhibition of S100A9 led to marked reduction in tissue injury and cell apoptosis. Consistent with the previous finding, the inflammatory responses were also significantly mitigated by blockade of S100A9. These findings supported the hypothesis that S100A9 could be considered as an important inflammatory mediator contributing to the progression of LPS-induced lung injury, and that inhibiting S100A9 could protect against LPS-induced lung injury in mice. However, a previous study reported that S100A9 attenuated acute lung injury, which was inconsistent with the results of the current study. This discrepancy could be due to the differences in the LPS stimulation process [32] or because in the current study the lung injury was observed 24 h following LPS injection, whereas in the previous one at 4 h. This discrepancy also suggests that S100A9 may exert immediate protective effects, and long-term elevation of S100A9 may boost inflammation and induce damage. Recent studies have reported that in COVID-19 patients the levels S100A9 are increased [33], suggesting that S100A9 may also play an important role in COVID-19-induced lung injury.

It has been demonstrated that excessive S100A9 accelerates the production of cytokines by macrophages and neutrophils, thus aggravating the inflammatory responses [34, 35]. These pro-inflammatory cytokines serve an important role in the activation of the inflammasome, which in turn increases the cleavage and production of cytokines [36], leading to a vicious cycle. Therefore, an association between S100A9 and inflammasome could not be ruled out. Herein, the blockade of S100A9 decreased the expression of NLRP3 in the lungs of LPS-treated mice. Furthermore, inhibition of S100A9 attenuated the production of inflammatory cytokines via inhibiting the NLRP3 pathway. These findings supported the aforementioned hypothesis regarding the association between S100A9 and inflammasome. A study demonstrated that inhibition of NLRP3 protected against LPS-induced lung injury [37]. In agreement with the above study, herein, lung injury was significantly mitigated in NLRP3^−/−^ mice. In addition, blockade of S100A9 could not provide additional protective effects in NLRP3^−/−^ mice. In vitro, NLRP3 overexpression blunted the effects of S100A9 inhibition on attenuating the production of proinflammatory cytokines, which further supported that the effects of S100A9 inhibition on LPS-induced lung injury could be associated with the suppression of the NLRP3 inflammasome activation.

It has been also reported that NLRP3 may cause apoptosis [38]. Therefore, the present study investigated the effects of S100A9 blockage on cell apoptosis in the lungs of LPS-treated mice. Bax protein serves an important role in regulating apoptosis by its redistribution from the cytosol to mitochondria [39], while caspase-3 is a crucial protease, mediating apoptotic cell death. The results of our study revealed that blockage of S100A9 downregulated Bax and caspase-3 expression in lung tissues, suggesting a blunted cell apoptosis in lungs. However, a recent study reported that apoptosis could also drive NLRP3 inflammasome assembly [40], indicating that Bax and caspase-3 could be involved in NLRP3 activation, and S100A9 inhibition could block the NLRP3 pathway via the expression of Bax and caspase-3. However, extensive research is needed to verify this hypothesis.

One limitation of the present study is that a pharmacological inhibitor was used to block S100A9, thereby, the non-target effects of the chemical inhibitor could not be excluded. Therefore, further verifying the results of the current study using a genetically modified mouse model is of great importance.

## Conclusions

In conclusion, the present study suggested that S100A9 could serve a key role in LPS-induced lung injury by triggering excessive inflammatory responses, partially via the NLRP3 pathway. Furthermore, S100A9 blockade alleviated lung injury in mice, thus providing a potential novel therapeutic target against acute lung injury.

## Data Availability

The datasets used and/or analyzed during the current study are available from the corresponding author on reasonable request.

## Abbreviations

ARDS: Acute respiratory distress syndrome
ABR: Quinoline-3-carboxamide analog
BALF: Bronchoalveolar lavage fluid acquisition
LPS: Lipopolysaccharide
LDH: Lactate dehydrogenase
MPO: Measurement of myeloperoxidase
